# Vaccination Coverage with Selected Vaccines and Exemption Rates Among Children in Kindergarten — United States, 2018–19 School Year

**DOI:** 10.15585/mmwr.mm6841e1

**Published:** 2019-10-18

**Authors:** Ranee Seither, Caitlin Loretan, Kendra Driver, Jenelle L. Mellerson, Cynthia L. Knighton, Carla L. Black

**Affiliations:** ^1^Immunization Services Division, National Center for Immunization and Respiratory Diseases, CDC; ^2^Association of Schools and Programs of Public Health Fellowship, Washington, DC; ^3^Oak Ridge Institute for Science and Education, Oak Ridge, Tennessee; ^4^Certified Technical Experts, Inc., Montgomery, Alabama.

State and local school vaccination requirements exist to ensure that students are protected against vaccine-preventable diseases (*1*). This report summarizes data collected by state and local immunization programs* on vaccination coverage among children in kindergarten in 49 states, exemptions for kindergartners in 50 states, and provisional enrollment and grace period status for kindergartners in 30 states. Nationally, vaccination coverage^†^ was 94.9% for the state-required number of doses of diphtheria and tetanus toxoids, and acellular pertussis vaccine (DTaP); 94.7% for 2 doses of measles, mumps, and rubella vaccine (MMR); and 94.8% for the state-required doses of varicella vaccine. Whereas 2.5% of kindergartners had an exemption from at least one vaccine,^§^ 2.8% of kindergartners were not up to date for MMR and did not have a vaccine exemption. Nearly all states could achieve the recommended ≥95% MMR coverage if all nonexempt kindergartners were vaccinated in accordance with local and state vaccination policies.

In accordance with state and local school entry requirements, parents and guardians submit children’s vaccination records or exemption forms to schools, or schools obtain records from state immunization information systems. Federally funded immunization programs collaborate with departments of education, school nurses, and other school personnel to assess vaccination coverage and exemption status of children enrolled in public and private kindergartens and to report unweighted counts, aggregated by school type, to CDC via a web-based questionnaire in the Secure Access Management System.^¶^ CDC uses these counts to produce state-level and national-level estimates of vaccination coverage. During the 2018–19 school year, 49 states reported coverage for all state-required vaccines among public school kindergartners; 48 states reported on private school kindergartners.** All 50 states reported exemption data among public school kindergartners; 49 states reported on private school kindergartners. Overall national and median vaccination coverage for the state-required number of doses of DTaP, MMR, and varicella vaccine are reported. Coverage with hepatitis B and poliovirus vaccines, which are required in most states but not included in this report, are available at SchoolVaxView (*2*). Thirty states reported data on kindergartners who, at the time of assessment, attended school under a grace period (attendance without proof of complete vaccination or exemption during a set interval) or provisional enrollment (school attendance while completing a catch-up vaccination schedule). Coverage and exemptions from the U.S. territories and affiliated jurisdictions are included in this report; however, national estimates, medians, and summary measures include only U.S. states.

Vaccination coverage and exemption estimates were adjusted according to survey type and response rates.^††^ For the 2018–19 school year, CDC is reporting national-level estimates alongside the state-level median estimates. The national estimates complement the medians by addressing the limitation that the median estimates weight every state equally regardless of population size. Reported estimates for the 2018–19 school year are based on 3,634,896 kindergartners surveyed for vaccination coverage, 3,643,598 for exemptions, and 2,813,482 for grace period and provisional enrollment among the 4,001,404 children reported as enrolled in kindergarten by the 50 state immunization programs.^§§^ Potentially achievable coverage with MMR, defined as the sum of the percentage of children up to date with 2 doses of MMR and those with no documented vaccination exemption but not up date, was calculated for each state. Nonexempt students include those provisionally enrolled, in a grace period, or otherwise without documentation of vaccination. SAS (version 9.4; SAS Institute) was used for all analyses.

Vaccination assessments varied by immunization program because of differences in states’ required vaccines and doses, vaccines assessed, methods, and data reported (Supplementary Table 1, https://stacks.cdc.gov/view/cdc/81811). Most states reported kindergartners as up to date for a given vaccine if they had received all doses of that vaccine required for school entry,^¶¶^ except seven states*** that reported kindergartners as up to date for any given vaccine only if they had received all doses of all vaccines required for school entry.

Nationally, 2-dose MMR coverage was 94.7% (range = 87.4% [Colorado] to ≥99.2% [Mississippi]). Coverage of ≥95% was reported by 20 states and coverage of <90% by two (Table). DTaP coverage was 94.9% (range = 88.8% [Idaho] to ≥99.2% [Mississippi]). Coverage of ≥95% was reported by 21 states, and coverage of <90% by one. Varicella vaccine coverage was 94.8% (range=86.5% [Colorado] to ≥99.2% [Mississippi]), with 20 states reporting coverage ≥95%, and four reporting <90% coverage.

**TABLE:** Estimated* vaccination coverage^†^ for measles, mumps, and rubella vaccine (MMR), diphtheria and tetanus toxoids and acellular pertussis vaccine (DTaP), and varicella vaccine, grace period or provisional enrollment,^§^ and any exemption^¶^ among children enrolled in kindergarten, by immunization program — United States, territories, and associated states, 2018–19 school year

Immunization program	Kindergarten population**	No. (%) surveyed^††^	MMR, 2 doses (%)^§§^	DTaP, 5 doses (%)^¶¶^	Varicella, 2 doses (%)***	Grace period or provisional enrollment (%)	Any exemption (%)	Percentage point change in any exemption from 2017 to 2018
**National estimate^†††^**	**4,001,404**	**3,634,896**	**94.7**	**94.9**	**94.8**	**2.0**	**2.5**	**0.2**
**Median^†††^**	**Not applicable**	**Not applicable**	**94.2**	**94.6**	**94.3**	**1.8**	**2.6**	**0.4**
**State**								
Alabama^§§§,¶¶¶^	77,739	77,739 (100.0)	≥90.6	≥90.6	≥90.6	NP	0.8	−0.1
Alaska^¶¶¶,^****	10,316	8,702 (84.4)	NR	NR	NR	NR	7.1	0.1
Arizona^§§§,††††^	79,981	79,981 (100.0)	92.9	92.7	95.6	NR	6.0	0.2
Arkansas^§§§§^	39,257	37,466 (95.4)	94.2	93.4	93.8	4.5	1.8	0.1
California^¶¶¶,††††,§§§§^	568,947	555,735 (97.7)	96.5	96.0	97.9	1.7	0.6	−0.1
Colorado^§§§,¶¶¶¶^	64,191	64,191 (100.0)	87.4	90.3	86.5	0.6	4.9	0.2
Connecticut^§§§,¶¶¶^	38,230	38,230 (100.0)	95.9	96.1	95.7	NP	2.7	0.4
Delaware^¶¶¶^	10,798	1,021 (9.5)	97.8	97.8	97.6	NR	1.2	−0.2
District of Columbia****	NA	NA	NR	NR	NR	NR	NR	NA
Florida^§§§,¶¶¶,^*****	224,641	224,641 (100.0)	≥93.8	≥93.8	≥93.8	2.9	3.2	0.3
Georgia^§§§,¶¶¶^	131,275	131,275 (100.0)	≥93.6	≥93.6	≥93.6	0.2	2.5	−0.2
Hawaii^¶¶¶^	16,051	1,081 (6.6)	91.5	92.4	94.0	1.3	4.4	1.3
Idaho	22,995	22,769 (99.0)	89.5	88.8	88.3	2.2	7.7	0.6
Illinois^§§§,¶¶¶¶^	143,876	143,876 (100.0)	94.7	94.7	94.4	1.1	1.8	0.2
Indiana^¶¶¶^	82,324	79,350 (96.4)	91.2	94.4	93.5	NR	1.3	0.4
Iowa^§§§,¶¶¶^	40,624	40,624 (100.0)	≥93.3	≥93.3	≥93.3	3.0	2.4	0.4
Kansas^¶¶¶,§§§§,†††††^	37,838	8,744 (23.1)	90.8	91.0	89.2	NR	2.1	0.4
Kentucky^¶¶¶,§§§§,^*****	55,587	55,024 (99.0)	93.4	94.1	92.8	NR	1.4	0.0
Louisiana^§§§^	56,203	56,203 (100.0)	95.5	97.7	95.4	NA	1.2	0.1
Maine	13,419	12,875 (95.9)	93.8	94.5	95.9	NR	6.2	0.9
Maryland^¶¶¶,§§§§^	71,431	71,423 (100.0)	97.4	97.7	97.1	NR	1.5	0.1
Massachusetts^§§§,¶¶¶,§§§§^	65,279	65,279 (100.0)	96.9	97.1	96.5	NP	1.4	0.1
Michigan^§§§^	118,632	118,632 (100.0)	94.6	94.8	94.3	0.6	4.5	0.3
Minnesota^¶¶¶¶,^*****	70,085	68,779 (98.1)	92.6	92.5	92.0	NR	3.7	0.2
Mississippi^§§§,¶¶¶,††††^	37,775	37,775 (100.0)	≥99.2	≥99.2	≥99.2	0.6	0.1	0.0
Missouri^§§§,¶¶¶¶^	72,687	72,687 (100.0)	94.8	94.8	94.5	NR	2.7	0.4
Montana^§§§,¶¶¶^	12,480	12,480 (100.0)	93.3	93.0	92.9	1.9	4.5	0.2
Nebraska^¶¶¶,§§§§,^	26,925	26,548 (98.6)	96.9	97.4	96.3	1.3	2.1	−0.1
Nevada^¶¶¶^	37,971	1,811 (4.8)	95.1	95.0	94.7	1.0	3.3	0.1
New Hampshire^¶¶¶^	12,421	12,421 (100.0)	≥91.8	≥91.8	≥91.8	4.9	3.3	0.4
New Jersey^§§§,¶¶¶^	109,161	109,161 (100.0)	≥95.0	≥95.0	≥95.0	1.1	2.5	0.3
New Mexico^¶¶¶^	25,269	25,170 (99.6)	96.1	96.0	95.7	1.9	1.5	−0.2
New York (including New York City)^§§§,¶¶¶^	220,495	220,495 (100.0)	97.2	96.7	96.7	1.9	1.3	0.2
New York City^§§§,¶¶¶^	96,912	96,912 (100.0)	97.7	97.0	97.1	1.2	0.7	0.0
North Carolina^¶¶¶,§§§§,^*****	124,343	113,074 (90.9)	93.2	93.2	93.1	1.6	1.6	−0.4
North Dakota	10,382	10,315 (99.4)	93.6	93.6	93.8	NR	4.3	0.9
Ohio	139,679	132,589 (94.9)	91.6	91.9	91.2	6.7	2.9	0.3
Oklahoma*****	54,806	50,456 (92.1)	92.2	92.7	95.8	NR	2.6	0.4
Oregon^§§§, §§§§^	45,870	45,870 (100.0)	93.0	92.4	94.3	NR	7.7	0.1
Pennsylvania	143,560	133,945 (93.3)	96.4	96.6	95.8	2.6	2.9	0.1
Rhode Island^§§§,¶¶¶,§§§§,^*****	10,964	10,964 (100.0)	97.4	97.4	97.0	NR	1.3	0.2
South Carolina^¶¶¶^	58,442	15,797 (27.0)	94.2	94.6	93.5	0.9	2.6	0.6
South Dakota^¶¶¶^	12,062	12,052 (99.9)	96.2	95.8	95.5	NR	2.6	0.4
Tennessee^§§§,¶¶¶,§§§§^	78,630	78,630 (100.0)	96.5	96.2	96.2	1.6	1.9	0.4
Texas (including Houston)^§§§§,^*****	390,034	387,530 (99.4)	96.9	96.7	96.5	1.5	2.4	0.4
Houston^§§§§,^*****	37,897	37,675 (99.4)	96.6	96.6	95.9	1.4	1.5	0.3
Utah^§§§^	50,179	50,179 (100.0)	92.8	92.4	92.5	2.3	5.7	0.4
Vermont^§§§,¶¶¶^	6,126	6,126 (100.0)	93.0	92.9	92.3	5.1	4.7	0.9
Virginia^¶¶¶,†††††^	100,394	4,422 (4.4)	95.0	98.0	93.6	NR	1.7	0.2
Washington*****	87,510	84,771 (96.9)	90.8	90.8	89.7	1.7	5.0	0.3
West Virginia^¶¶¶,††††,§§§§§^	19,442	15,426 (79.3)	98.8	98.7	98.5	2.3	0.8	0.6
Wisconsin^§§§§,^*****^,†††††^	66,344	1,530 (2.3)	92.6	96.2	91.6	4.9	5.9	0.5
Wyoming	7,734	7,734 (100.0)	95.1	95.3	94.7	2.5	2.9	NA
**Territories and associated states**								
American Samoa^¶¶¶^	NA	NA	NA	NA	NReq	NP	NA	NA
Federated States of Micronesia^§§§^	1,786	1,786 (100.0)	91.3	80.2	NReq	NR	0.0	0.0
Guam^¶¶¶^	2,563	735 (28.7)	88.4	90.7	NReq	NR	0.1	-0.3
Marshall Islands^§§§,¶¶¶,††††^	1,114	1,114 (100.0)	95.1	83.8	NReq	NR	0.0	0.0
Northern Mariana Islands^§§§^	812	812 (100.0)	97.7	79.4	98.2	NR	0.0	0.0
Palau^§§§,¶¶¶¶¶^	304	304 (100.0)	100.0	100.0	NReq	NR	0.0	0.0
Puerto Rico	26,353	1,545 (5.9)	94.7	91.4	94.7	NR	1.6	NA
U.S. Virgin Islands	NA	NA	NA	NA	NA	NA	NA	NA

**Abbreviations:** NA = not available; NP = no grace period/provisional policy; NR = not reported to CDC; NReq = not required.

* Estimates are adjusted for nonresponse and weighted for sampling where indicated.

^†^ Estimates based on a completed vaccine series (i.e., not vaccine-specific) use the “≥” symbol. Coverage might include history of disease or laboratory evidence of immunity.

^§^ A grace period is a set number of days during which a student can be enrolled and attend school without proof of complete vaccination or exemption. Provisional enrollment allows a student without complete vaccination or exemption to attend school while completing a catch-up vaccination schedule. In states with one or both of these policies, the estimates represent the number of kindergartners within a grace period, provisionally enrolled, or some combination of these categories.

^¶^ Exemptions, grace period, provisional enrollment, and vaccine coverage status might not be mutually exclusive. Some children enrolled under a grace period or provisional enrollment might be exempt from one or more vaccinations, while children with exemptions might be fully vaccinated with one or more required vaccines.

** The kindergarten population is an approximation provided by each program. The national total excludes the 8,075 kindergartners from the District of Columbia for which data were not reported.

^††^ The number surveyed represents the number of kindergartners surveyed for vaccination coverage. For Alaska, this number represents the number surveyed for exemptions because coverage was not reported. The national total excludes the 8,702 kindergartners from Alaska. Exemption estimates are based on 31,792 kindergartners for Kansas, 95,875 kindergartners for Virginia, and 66,652 kindergartners for Wisconsin.

^§§^ Most states require 2 doses of MMR; Alaska, New Jersey, and Oregon require 2 doses of measles, 1 dose of mumps, and 1 dose of rubella vaccines. Georgia, New York, New York City, North Carolina, and Virginia require 2 doses of measles and mumps, 1 dose of rubella vaccines. Iowa requires 2 doses of measles and 2 doses of rubella vaccines.

^¶¶^ Pertussis vaccination coverage might include some diphtheria, tetanus toxoids, and pertussis vaccine (DTP) vaccinations if administered in another country or by a vaccination provider who continued to use DTP after 2000. Most states require 5 doses of DTaP for school entry, or 4 doses if the 4th dose was received on or after the 4th birthday; Illinois, Maryland, Virginia, and Wisconsin require 4 doses; Nebraska requires 3 doses. The reported coverage estimates represent the percentage of kindergartners with the state-required number of DTaP doses, except for Kentucky, which requires ≥5 doses but reports ≥4 doses of DTaP.

*** Most states require 2 doses of varicella vaccine for school entry; Alabama, Arizona, California, Hawaii, Maine, New Jersey, Oklahoma, and Oregon require 1 dose. Reporting of varicella vaccination status for kindergartners with a history of varicella disease varied within and among states; some were reported as vaccinated against varicella and others as medically exempt.

^†††^ National coverage estimates and medians calculated from data from 49 states (i.e., does not include Alaska). National grace period or provisional enrollment estimate and median were calculated from data from 30 states that have either a grace period or provisional enrollment policy and reported relevant data to CDC. National exemption estimate and median were calculated from data from 50 states. Other jurisdictions excluded were Houston, Texas, New York City, American Samoa, Guam, Marshall Islands, Federated States of Micronesia, Northern Mariana Islands, Palau, Puerto Rico, and U.S. Virgin Islands. Data reported from 3,634,896 kindergartners assessed for coverage, 3,643,598 for exemptions and 2,813,482 for grace period/provisional enrollment. Estimates represent rates for populations of 3,991,088; 4,001,404; and 3,025,009 kindergartners for coverage, exemptions and grace period/provisional enrollment, respectively.

^§§§^ The proportion surveyed likely was <100% but is reported as 100% based on incomplete information about the actual current enrollment.

^¶¶¶^ Philosophical exemptions were not allowed.

**** Kindergarten vaccination coverage (Alaska and District of Columbia) and exemption data (District of Columbia) were not reported because of problems with data collection.

^††††^ Religious exemptions were not allowed.

^§§§§^ Counted some or all vaccine doses received regardless of Advisory Committee on Immunization Practices recommended age and time interval; vaccination coverage rates reported might be higher than those for valid doses.

^¶¶¶¶^ Program did not report the number of children with exemptions, but instead reported the number of exemptions for each vaccine, which could count some children more than once. Lower bounds of the percentage of children with any exemptions were estimated using the individual vaccines with the highest number of exemptions.

***** Did not include some types of schools, such as online schools or those located on military bases, in correctional facilities, or on tribal lands.

^†††††^ Kindergarten vaccination coverage data were collected from a sample, and exemption data were collected from a census of kindergartners.

^§§§§§^ Reported public school data only.

^¶¶¶¶¶^ For Palau, estimates represent coverage among children in first grade.

The percentage of kindergartners with an exemption from one or more required vaccines (not limited to MMR, DTaP, and varicella vaccines) was 2.5% in 2018–19 (range = 0.1% [Mississippi] to 7.7% [Idaho and Oregon]). This is slightly higher than the 2.3% during the 2017–18 school year and 2.1% in 2016–17. (Table) (Figure 1). Nationally, 0.3% of kindergartners had a medical exemption, and 2.2% had a nonmedical exemption (Supplementary Table 2, https://stacks.cdc.gov/view/cdc/81810).

**FIGURE 1:**
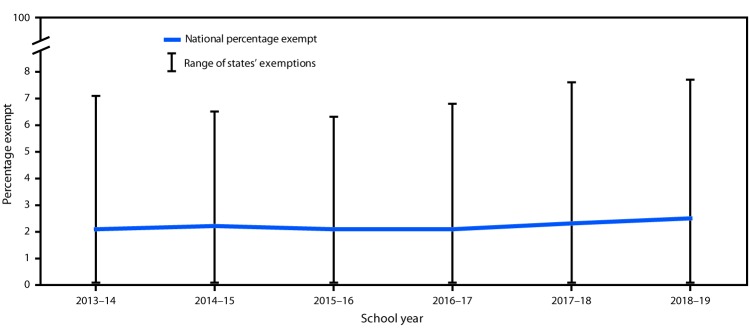
Estimated national percentage exempt and range of states’ exemptions from one or more vaccines among kindergartners — United States, 2013–14 to 2018–19 school years

The percentage of kindergartners attending school within a grace period or provisionally enrolled among the 30 states reporting these data was 2.0% (range = 0.2% [Georgia] to 6.7% [Ohio]) (Table). In 10 of these states, the percentage of children provisionally enrolled or within a grace period at the time of assessment exceeded the percentage of children with exemptions from one or more vaccines. Forty-four states could potentially achieve ≥95% MMR coverage if all nonexempt kindergartners, many of whom are within a grace period or provisionally enrolled, were vaccinated (Figure 2). Follow-up could assure all missing vaccinations are completed and all missing documentation of vaccination is provided to schools.

**FIGURE 2:**
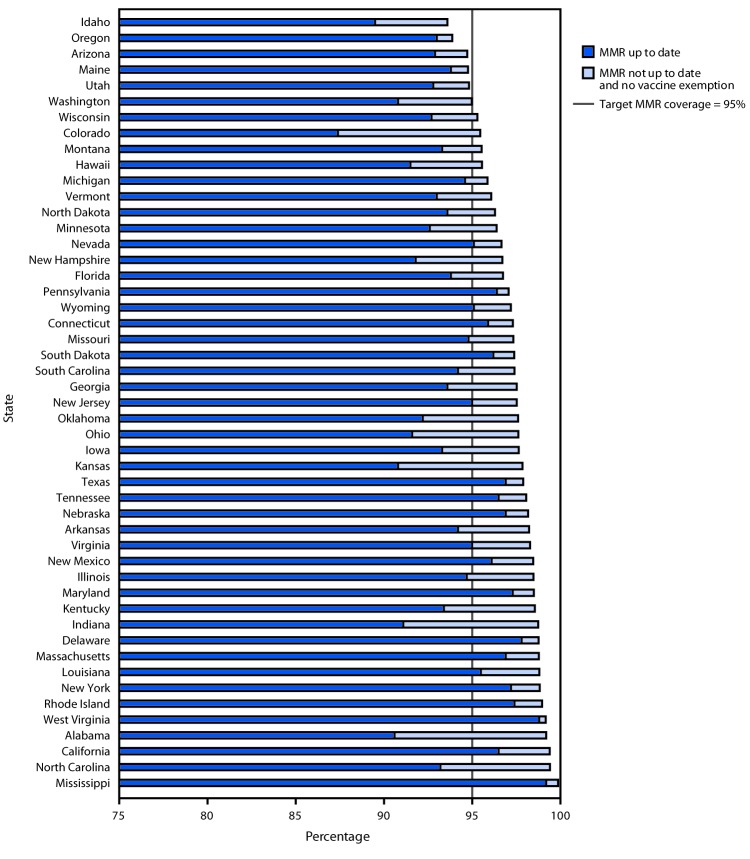
Potentially achievable coverage*^,†,§^ with measles, mumps, and rubella vaccine (MMR) among kindergartners — 49 states, 2018–2019 school year * Potentially achievable coverage is estimated as the sum of the percentage of students with up-to-date MMR and the percentage of students without up-to-date MMR and without a vaccine exemption. ^†^ The exemptions used to calculate the potential increase in MMR coverage for Arizona, Arkansas, Colorado, Idaho, Illinois, Maine, Massachusetts, Michigan, Minnesota, Missouri, Nebraska, New York, North Carolina, North Dakota, Ohio, Oklahoma, Oregon, Rhode Island, Texas, Utah, Vermont, and Wyoming are the number of children with exemptions specifically for MMR vaccine. For all other states, numbers are based on an exemption to any vaccine. ^§^ Alaska and the District of Columbia did not report kindergarten vaccination coverage for the 2018–19 school year and are excluded from this analysis.

## Discussion

Measles outbreaks affecting school-age children across multiple states during the 2018–19 school year underscore the importance of both school vaccination requirements for preventing disease spread and school coverage assessments to identify pockets of undervaccination (*3*). During the 2018–19 school year, national coverage with MMR, DTaP, and varicella vaccines remained near 95% (*2*,*4*). However, coverage and exemption rates varied by state. Recent measles outbreaks in states with high overall MMR coverage, such as New York, highlight the need for assessing vaccination coverage at the local level. CDC encourages programs to use their local-level school assessment data to identify populations of undervaccinated students and to partner with schools and providers to reduce barriers to vaccination and improve coverage.

Although the overall percentage of children with an exemption increased slightly for the second consecutive school year, children with exemptions still represent a small proportion of kindergartners nationally and in most states. More importantly, in 25 states, the number of nonexempt undervaccinated kindergartners exceeded the number of those with exemptions. In many states, nonexempt undervaccinated students are attending school in a grace period or are provisionally enrolled. Fifteen states allow grace periods, with 30 days the most common length, and 47 states allow provisional enrollment for students in the process of completing the vaccination schedule (R McCord, CDC, unpublished data, 2019). Follow-up with parents of these students to verify that vaccinations and related documentation are complete typically falls to school nurses or other school staff members (R Seither, CDC, unpublished data, 2019). The California Department of Public Health’s immunization program collaborated with the state Department of Education and with individual schools to reduce provisional enrollment substantially over several years, which resulted in measurable increases in vaccination coverage, through training on the correct application of the relevant rules so that only those children who were completing a catch-up schedule were provisionally enrolled, and audits to assess the implementation by school staff members (*5*,*6*). Almost all states could achieve ≥95% MMR coverage if undervaccinated nonexempt children were vaccinated in accordance with local and state vaccination policies.

The findings in this report are subject to at least five limitations. First, comparability is limited because of variation in states’ requirements, data collection methods, and definitions of grace period and provisional enrollment. Second, representativeness might be negatively affected because of data collection methods that miss some schools or students, such as homeschooled students, or assess vaccination status at different times. Third, actual vaccination coverage, exemption rates, or both might be underestimated or overestimated because of inaccurate or absent documentation or missing schools. Fourth, national coverage estimates include only 49 of 50 states, exemption estimates include all states but use lower-bound estimates for four states, and grace period or provisional enrollment estimates include only 30 states for the 2018–19 school year. Finally, because most states do not report vaccine-specific exemptions, estimates of potentially achievable MMR coverage are approximations. However, if reported exemptions were for a vaccine or vaccines other than MMR, potentially achievable MMR coverage would be higher than that presented.

Kindergarten vaccination requirements help ensure that students are fully vaccinated with recommended vaccines upon school entry. CDC works with immunization programs to collect and report data on school vaccination coverage, exemption rates, and grace period and provisional enrollment each year. Immunization programs can use these data to identify schools and communities with high concentrations of undervaccinated students and inform strategies to increase vaccination coverage. Such strategies include education campaigns to counteract misinformation in areas with high numbers of vaccine exemptions and increased follow-up of undervaccinated students without exemptions to ensure these children are vaccinated in accordance with local and state vaccination policies (*7*) to reduce the risk for transmission of vaccine-preventable diseases.

**Summary**

**What is already known about this topic?**

State immunization programs conduct annual kindergarten vaccination assessments to monitor school-entry vaccination coverage with all state-required vaccines.

**What is added by this report?**

For the 2018–19 school year, coverage was 94.7% for 2 doses of measles, mumps, and rubella vaccine (MMR) and 94.9% for the state-required number of doses of diphtheria and tetanus toxoids and acellular pertussis vaccine, and 94.8% for varicella vaccine. Although the exemption rate slightly increased to 2.5%, most states could achieve the recommended ≥95% MMR coverage if undervaccinated children without an exemption were completely vaccinated.

**What are the implications for public health practice?**

State and local immunization programs can use school coverage assessments to detect pockets of undervaccination and guide strategies to increase vaccination coverage.

## Footnotes

* Federally funded immunization programs are located in the 50 states and the District of Columbia (DC), five other cities, and eight U.S territories and freely associated states (territories). Two cities reported data to CDC, which were also included in data by their state, to calculate medians and national estimates. Immunization programs in U.S. territories reported vaccination coverage and exemptions to CDC; however, these data were not included in overall national calculations.

† National and median vaccination coverage was determined using estimates for 49 states; Alaska and DC did not report school coverage data because of problems with data collection. Data from cities were included with their state data. Data from territories were not included in national and median calculations.

§ National and median exemption rates were determined using estimates for all 50 states; Colorado, Illinois, Minnesota, and Missouri did not collect information on the number of kindergartners with an exemption but instead reported the number of exemptions for each vaccine, which could count some children more than once. For these states, the percentage of kindergartners exempt from the vaccine with the highest number of exemptions (the lower bound of the potential range of exemptions) was included in the national and median exemption rates. DC did not report school exemption data because of problems with data collection. Data from cities were included with their state data. Data from territories were not included in national estimates.

¶ Assessment date varied by state and area. Six states assess on the first day of school; 16 states assess by December 31; 14 states assess by some other date, ranging from 30 days after admission to April 25; 14 states assess on a rolling basis.

** Nine states reported coverage and exemption data for at least some homeschooled kindergartners. California included data for 17 independent study schools and at least two virtual schools registered with the California Department of Education in public school data, and data for homeschools with six or more students in private school data. Delaware reported the only documented homeschool in the state within private school data. New Mexico included students from one public online academy in public school data. North Dakota reported some homeschool data separately. Oregon reported a convenience sample of homeschooled students enrolled through online public schools separately; some children enrolled in public online homeschools were included in the public school data. Pennsylvania included all homeschooled students in their public school data. Utah included students enrolled in public online academies in public school data, and students enrolled in private online academies in private school data. Vermont included homeschooled students in their public and private school data if the students were enrolled in one or more classes at a school. Wyoming reported at least some homeschooled students in public school data.

†† Most immunization programs that used census or voluntary response provided CDC with data aggregated at the state or local (city or territory) level. Coverage and exemption data based on a census or voluntary response were adjusted for nonresponse using the inverse of the response rate, stratified by school type (public, private, and homeschool, where available). Programs that used complex sample surveys provided CDC with deidentified data aggregated at the school or county level for weighted analysis. Weights were calculated to account for sample design and adjusted for nonresponse for data collected through complex sample design wherever possible.

§§ The totals reported here are the summations of the kindergartners surveyed among programs reporting data for coverage, exemptions, grace periods, and provisional enrollment. Data from cities and territories were not included in these totals.

¶¶ All states required 2 doses of a measles-containing vaccine. Local DTaP requirements varied. Nebraska required 3 doses, four states (Illinois, Maryland, Virginia, and Wisconsin) required 4 doses, and all other states required 5 doses, unless the 4th dose was administered on or after the fourth birthday. The reported coverage estimates represent the percentage of kindergartners with the state-required number of DTaP doses, except for Kentucky, which required 5 doses of DTaP by age 5 years but reported 4-dose coverage for kindergartners. Seven states required 1 dose of varicella vaccine; 44 states required 2 doses.

*** Alabama, Florida, Georgia, Iowa, Mississippi, New Hampshire, and New Jersey considered kindergartners up to date only if they had received all doses of all vaccines required for school entry.
